# Dynamic acetylation profile during mammalian neurulation

**DOI:** 10.1002/bdr2.1618

**Published:** 2019-11-23

**Authors:** Valentina Massa, Laura Avagliano, Paolo Grazioli, Sandra C. P. De Castro, Chiara Parodi, Dawn Savery, Patrizia Vergani, Serena Cuttin, Patrizia Doi, Gaetano Bulfamante, Andrew J. Copp, Nicholas D. E. Greene

**Affiliations:** ^1^ Department of Health Sciences University of Milan Milan Italy; ^2^ UCL Great Ormond Street Institute of Child Health University College London London UK; ^3^ Department of Obstetrics and Gynaecology, Foundation MBBM University of Milano‐Bicocca Monza Italy; ^4^ Department of Pathology, San Gerardo Hospital University of Milano‐Bicocca Monza Italy

**Keywords:** acetylation profile, *Cited2*, neural tube defects, neurodevelopment, p53

## Abstract

**Background:**

Neural tube defects (NTDs) result from failure of neural tube closure during embryogenesis. These severe birth defects of the central nervous system include anencephaly and spina bifida, and affect 0.5–2 per 1,000 pregnancies worldwide in humans. It has been demonstrated that acetylation plays a pivotal role during neural tube closure, as animal models for defective histone acetyltransferase proteins display NTDs. Acetylation represents an important component of the complex network of posttranslational regulatory interactions, suggesting a possible fundamental role during primary neurulation events. This study aimed to assess protein acetylation contribution to early patterning of the central nervous system both in human and murine specimens.

**Methods:**

We used both human and mouse (*Cited2* ^*−/−*^) samples to analyze the dynamic acetylation of proteins during embryo development through immunohistochemistry, western blot analysis and quantitative polymerase chain reaction.

**Results:**

We report the dynamic profile of histone and protein acetylation status during neural tube closure. We also report a rescue effect in an animal model by chemical p53 inhibition.

**Conclusions:**

Our data suggest that the p53‐acetylation equilibrium may play a role in primary neurulation in mammals.

## 1 INTRODUCTION

The neural tube is the embryonic precursor of the brain and spinal cord, and formation of this structure is a critical process in embryonic development. In humans, neural tube closure occurs during the third and fourth weeks after conception. Neural tube defects (NTDs) arise if closure is not completed, and the neuroepithelium remains vulnerable to damage as it is not covered or protected. The most common open NTDs include anencephaly and spina bifida (Avagliano et al., 2018). Neurulation requires the coordinated function of many gene products, as revealed by the large number (more than 290) of genetic mouse mutant strains in which closure fails, resulting in NTDs (Copp, Greene, & Murdoch, 2003; Harris & Juriloff, 2007). Defects in mice closely parallel the corresponding severe birth defects in humans which affect 0.5–2 per 1,000 pregnancies (Greene & Copp, 2014; Wallingford, Niswander, Shaw, & Finnell, 2013). Although mouse mutants implicate a large number of genes in neural tube closure, the molecular basis of NTDs is not well understood in the majority of human cases.

Acetylation is a post‐translational modification occurring on histones and non‐histone proteins that is capable of regulating protein functions, localization, and stability (Narita, Weinert, & Choudhary, 2018). A potential requirement for acetylation in neural tube closure has been suggested by the observation of cranial NTDs in mouse embryos that carry even one null allele of *p300*, a transcriptional co‐activator that has potent histone acetyltransferase (HAT) activity (Harris & Juriloff, 2007). Interestingly, NTDs also occur at high frequency in null embryos for *Cited2* (Barbera et al., 2002), a CBP/P300‐interacting transactivator protein containing a Glu/Asp‐rich C‐terminal domain. Null embryos for the acetyltransferase gene *Gcn5*, die early in gestation with developmental delay and extensive apoptosis in mesodermal lineages (Bu, Evrard, Lozano, & Dent, 2007; Xu et al., 2000). Double mutants of *Gcn5* and *p53* survive longer and have reduced levels of apoptosis but they still do not develop to the stage of neural tube closure (Bu et al., 2007). A knock‐in allele: *Gcn5* ^*hat*^, was generated which incorporates point mutations in the catalytic domain that result in specific ablation of HAT activity. Homozygous *Gcn5* ^*hat/hat*^ embryos exhibit cranial NTDs, without growth retardation or excessive apoptosis (Lin et al., 2008). Thus, both decreased acetylation (HAT mutants) and increased acetylation (*Cited2*) might be associated with the development of NTDs. A possible role for disturbed acetylation in NTDs also arises from consideration of possible mechanisms underlying teratogenic effects of antiepileptic drugs such as valproic acid (VPA), whose use during pregnancy is a major risk factor for spinal NTDs in humans (Wlodarczyk, Palacios, George, & Finnell, 2012) and in animal models (Finnell, 1991; Hughes, Greene, Copp, & Galea, 2018). VPA has efficient histone deacetylase (HDAC) inhibitor activity, especially in embryonic tissue susceptible to its toxicity (Menegola et al., 2005).

In summary, acetylation represents an important component of the complex network of post‐translational regulatory interactions. Evidence from mouse models indicates a possible fundamental role during primary neurulation events. It is assumed that acetylation also occurs during human development (Goodman & Smolik, 2000), but is not known whether this alters with stage or is affected in NTDs. In mice, the overall acetylation status during neurulation stages has not been evaluated. Hence, the aim of this study was to assess protein acetylation in the context of early development of the mouse and human central nervous system.

## 2 MATERIALS AND METHODS

### 2.1 Human samples

#### 2.1.1 Human cohort

Controls: formalin fixed, paraffin‐embedded samples were obtained from four healthy Caucasian women undergoing legal termination of singleton pregnancy from 9 to 26 weeks of gestation (WG). Samples were obtained from archival tissues from the Unit of Human Pathology of the San Paolo Hospital Medical School, Milano, Italy. In this group, fetuses did not exhibit any malformations. The NTD case was from legal termination at 21 WG of a singleton pregnancy affected by spina bifida in a Caucasian woman with epilepsy who was treated with VPA. Termination of pregnancy occurred at Department of Obstetrics and Gynecology, Foundation MBBM, Monza, Italy.

#### 2.1.2 Immunohistochemistry

Immunohistochemistry was performed as previously described (Avagliano et al., 2016) on 5 μm sections of paraffin‐embedded tissue using a Ventana system (Ventana Medical Systems, Tucson, AZ) according to the manufacturer's instructions. The primary antibody was specific for acetyl‐histone H4 (Acetyl‐Histone H4 (Lys5) (D12B3) Rabbit mAb #8647, Cell Signaling). Staining was achieved using Ultraview Universal DAB detection kit (Ventana Medical Systems) and counterstained with hematoxylin. One fetus per stage was selected for this study. Slides were immunostained in the same batch to prevent technical variability and ensure identical conditions for comparison.

#### 2.1.3 Counting

Images were digitalized and captured using a NanoZoomer‐XR Digital slide scanner (Hamamatsu, Japan). After scanning the entire section, five randomly selected fields of view per case and controls were photographed at ×20 magnification and analyzed using ImageJ 1.47v software (Bethesda, MD). Images were calibrated with a stage micrometer. H4 positive cells were calculated by counting within the five fields, by three operators blinded to experimental groups.

### 2.2 Animal studies

#### 2.2.1 Animals

Animal studies were carried out under regulations of the Animals (Scientific Procedures) Act 1986 of the UK Government, and according to guidance issued by the Medical Research Council, United Kingdom, in Responsibility in the Use of Animals for Medical Research (July 1993). Random‐bred CD1 mice were purchased from Charles River Laboratories, United Kingdom. *Cited2* mutant, heterozygous and corresponding wild‐type embryos were obtained from four litters in each experimental group and genotyped as previously reported (Barbera et al., 2002). Animals were paired overnight and females checked for vaginal plugs the following morning, designated embryonic day (E) 0.5. Embryos were dissected from the uterus at desired developmental stages, morphologically assessed, somites counted for staging, and frozen at −80°C for further analyses. Pifithrin‐α (Komarov et al., 1999) (2 mg/kg) or vehicle (PBS and saline) was administered by intraperitoneal (i.p.) injection at E7.5, E8.5, and E9.5.

#### 2.2.2 Western blot

Total proteins were extracted from embryos by standard procedures (*n* = 3 embryos per pool per genotype). Following protein quantification, equal amounts of quantified extracts were used for western blot analyses as previously described (de Castro et al., 2012) using antibodies against acetylated lysines (Acetylated‐Lysine Antibody #9441, Cell Signaling) or acetylated‐p53 (Acetyl‐p53 [Lys379‐specific for mouse] Antibody #2570, Cell Signaling). Positive bands were quantified, and analyses were performed using GraphPad.

#### 2.2.3 Quantitative polymerase chain reaction

RNA was extracted with TRIzol (Invitrogen, United Kingdom) as previously described (Fazio et al., 2017). First strand cDNA was synthesized using a Superscript first‐strand Synthesis system (Invitrogen) following manufacturer's protocol. GreenER qPCR Supermix (Invitrogen) with Biotaq DNA polymerase (Bioline, United Kingdom) was used for quantitative polymerase chain reaction (qPCR) analyses on a Fast System 7500 with SDS system software (Applied Biosystems). Primers used were:

p53_Left: GCTTCTCCGAAGACTGGATG

p53_Right: CTTCACTTGGGCCTTCAAAA

GAPDH_Left: ATGACATCAAGAAGGTGGTG

GAPDH_Right: CATACCAGGAAATGAGCTTG

#### 2.2.4 Data analysis

For human data, acetyl‐histone H4 positive cells were counted as described and Mann–Whitney test was used to compare cases and controls. For animal studies, *t* test on qPCR data was applied. In both cases *p* ≤ 0.05 was set as significant. GraphPad 6 software (San Diego, CA) and Photoshop (Adobe Photoshop CC) software were utilized for data analysis and figure preparation.

## 3 RESULTS

**Figure 1:**
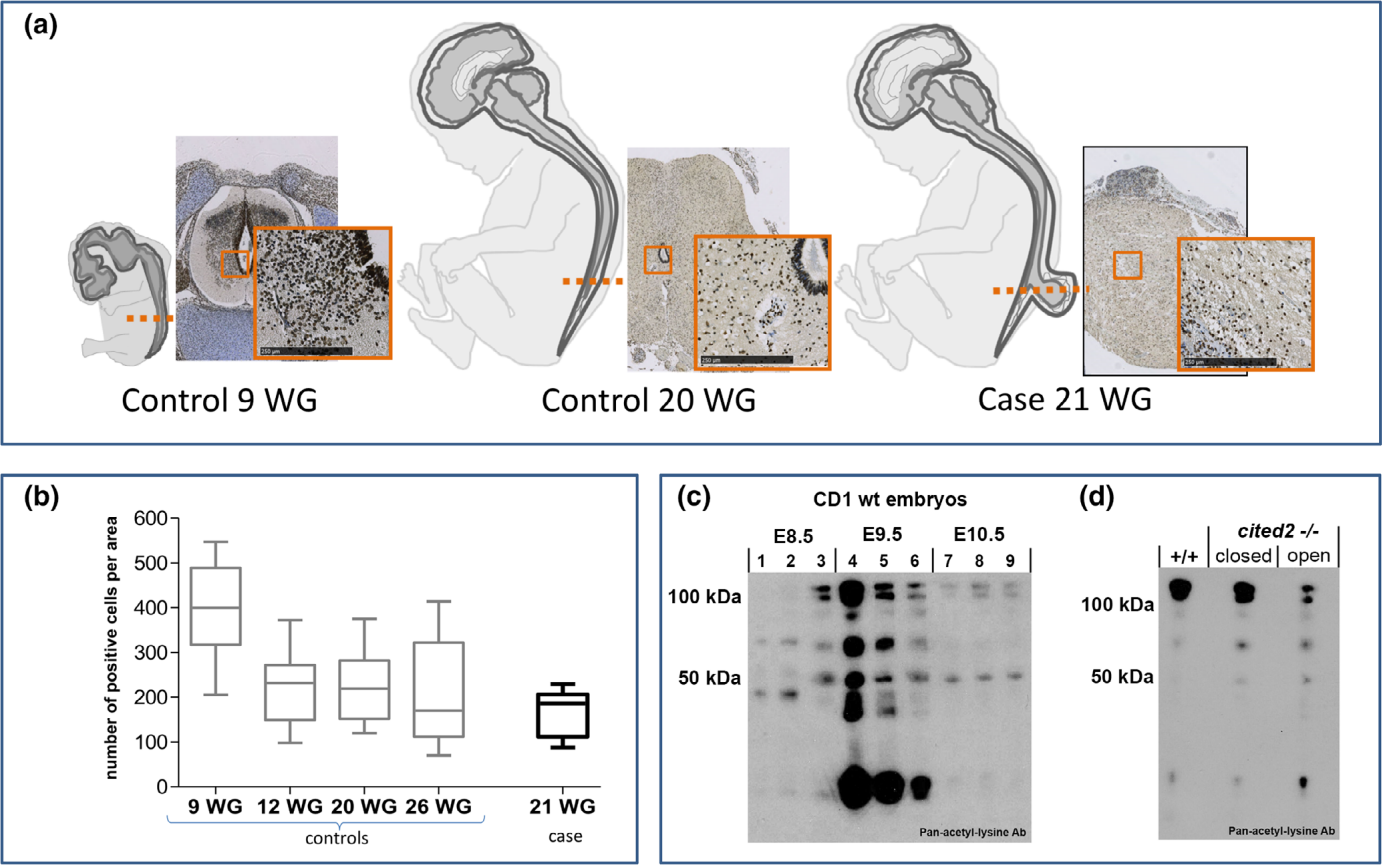
Acetylation status is dynamic during human and mouse neural tube development. (a) Cells staining for acetylated‐histone H4 antibody are present in the normal developing human neural tube (Control), both at 9 and 20 WG. Note positive‐staining cells also in the spinal cord of a 21 WG individual with spina bifida (Case). Bar represents 250 μm. The orange line represent the level of histological section (lumbar for all analyzed fetuses). (b) Quantification of acetylated‐histone H4 staining: the median value is shown as a horizontal line inside the boxes; upper and lower limits of the boxes represent upper and lower quartiles, respectively; whiskers represent the maximum and minimum values. Among normal fetuses (controls), an early stage of gestation (9 WG) has a higher number of acetylated‐histone H4 positive cells per unit area than stages from 12 WG onward. The fetus with spina bifida (case) shows a similar density of positive cells as unaffected individuals of a comparable stage. (c) Western blot demonstrates the dynamic acetylation profile of wild‐type mouse embryos at different developmental stages. Note the band at 50 KDa which has a peak of expression intensity at E9.5. (d) The acetylation profile of wild‐type and *Cited2* ^*−/−*^ embryos without (closed) and with (open) exencephaly. A 50 KDa band is upregulated in *Cited2* ^*−/−*^ embryos compared with wild‐type, with particularly strong acetylation in mutants with open neural tube

To assess the overall histone acetylation profile during early human spinal cord development, immunohistochemical analysis was used to enable counting of acetylated‐histone H4 positive cells. At 9 WG, many positive cells could be detected in the developing spinal cord (Figure 1a,b). The density of acetylated‐histone H4 expressing cells decreased at 12 WG, and then remained constant until at least 26 WG (Figure 1a,b). An NTD‐affected fetus (open spina bifida following VPA exposure, 21 WG) displayed a similar density of positive cells as controls of comparable developmental stages.

Neural tube closure events occur at earlier stages of development, 3–4 weeks, than in this group of human fetuses. Therefore, for an assessment of the acetylation profile during neurulation we turned to the mouse, in which the neurulation process is similar to humans. In wild‐type embryos, global protein acetylation levels were compared, using an antibody to acetylated lysine, shortly after initiation of neural tube closure (E8.5), during cranial and upper spinal closure (E9.5), and during the final stages of closure when the low spine is formed (E10.5). This analysis showed a dynamic pattern (Figure 1c), with an overall increase of protein acetylation at E9.5. Intriguingly some protein bands (e.g., 50 K protein in Figure 1c) showed a distinct up‐regulation of acetylation status at E9.5, showing a temporal correlation with neurulation events.

We asked whether the observed changes in acetyl‐lysine staining are likely to reflect differential regulation of acetylation. For this analysis, we used *Cited2* mutants as a positive control. Cited2 interacts with the known HAT, p300, hence we studied the acetylation profile of mouse embryos from litters carrying a loss‐of‐function mutation in *Cited2* (Bhattacharya et al., 1999). Embryo protein samples from E10.5 wild‐type embryos were compared with those from littermate *Cited2* null embryos that were either unaffected (closed brain) or with an NTD (open brain; exencephaly). Intriguingly, a consistent difference in acetylation profile was observed in the three pools of embryos. In particular, a band at around 50 KDa, was absent from wild‐type extracts, became visible in unaffected *Cited2* ^−/−^ embryos, and appeared strongly acetylated in *Cited2* ^−/−^ embryos in which neural tube closure had recently failed (Figure 1d).

**Figure 2:**
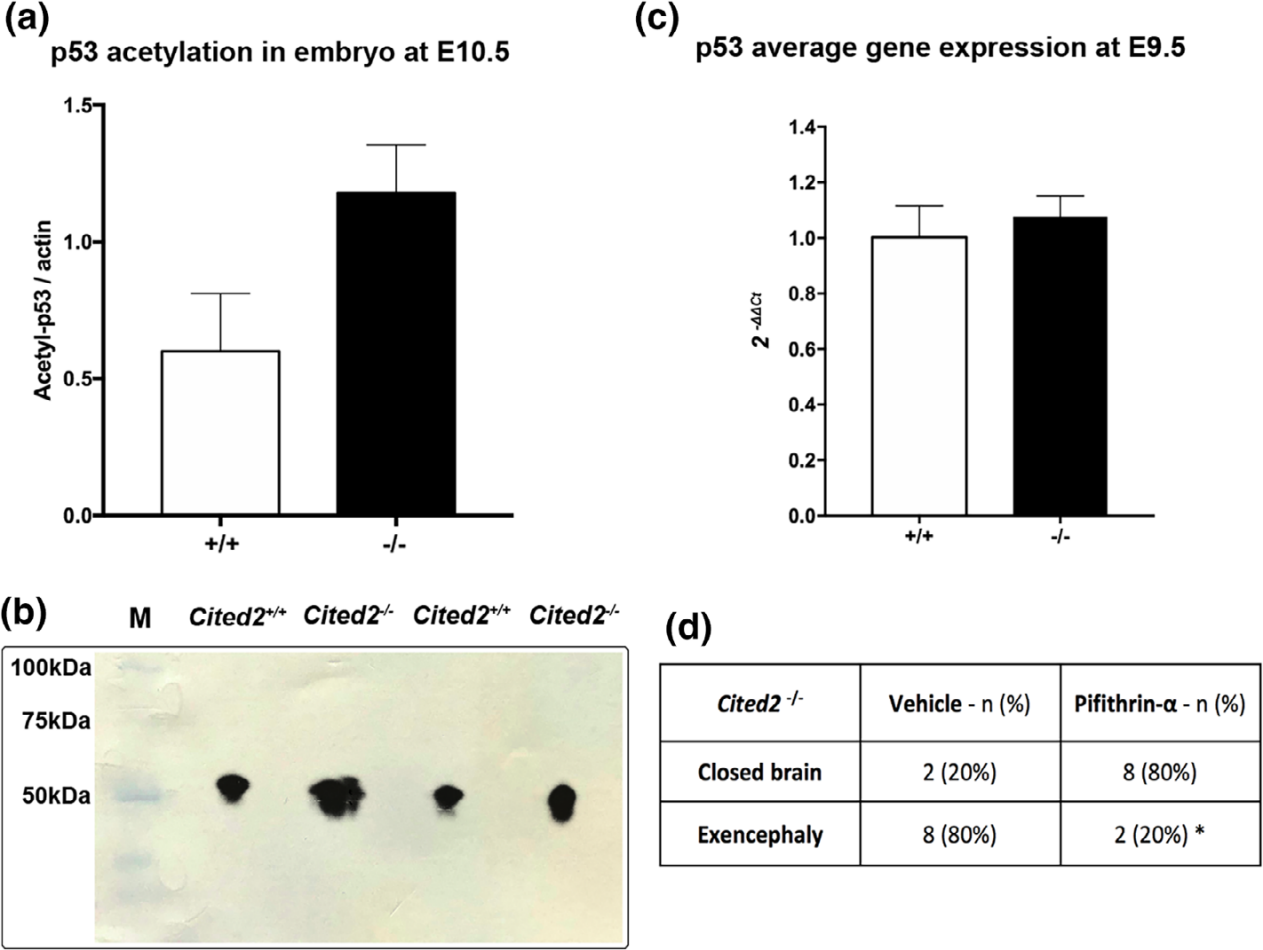
p53 acetylation contribution to the *Cited2* ^−/−^ NTD phenotype. (a) Quantification of acetylated p53, normalized to actin expression, shows an increase in affected *Cited2* ^*−/−*^ embryos (three embryos per pool per genotype were used. Three replicates were performed). Bars show standard deviation. (b) Relative western blot analysis of acetylated p53. (c) In contrast, total *p53* gene expression (qPCR) does not differ between *Cited2* genotypes at E9.5. (d) Chemical inhibition of p53 activity using pifithrin‐α results in a significant decrease in the frequency of NTDs in *Cited2* ^*−/−*^ embryos (Fisher's exact test: *p = 0.023)

Considering the importance of p53 in cell death and differentiation during embryonic development (Van Nostrand, Bowen, Vogel, Barna, & Attardi, 2017), and the requirement for acetylation to prevent p53 degradation (Reed & Quelle, 2014), we explored the possibility that p53 is itself acetylated to a greater extent in affected *Cited2* ^−/−^ mutants compared with wild‐type embryos. Protein extracts from wild‐type and affected *Cited2* ^−/−^ embryos were used for western blot analysis of acetylated‐p53 and, a 30% increase in normalized band intensity was found in mutant samples (Figure 2a). Analysis of *Trp53* mRNA abundance found no significant difference between wild‐type, *Cited2* ^+/−^ and *Cited2* ^−/−^ embryos at E9.5 (Figure 2b), showing that p53 abundance was not misregulated at the transcriptional level. Hence, acetylated‐p53 shows a specific increase in abundance in affected *Cited2* mutant embryos.

We explored the possible functional relevance of p53 activity in the causation of NTDs in this mouse model. Pregnant *Cited2* ^+/−^ females were treated by i.p. injection either with vehicle (PBS and saline) or with a chemical compound known to selectively inhibit p53 activity (pifithrin‐α) at three time‐points (E7.5, E8.5, and E9.5). On morphological evaluation of litters at E10.5, pifithrin‐α treatment was found to result in a significant reduction in the frequency of NTDs in *Cited2* ^−/−^ embryos. Hence, while 80% of control mutant embryos had exencephaly, only 20% of pifithrin‐α treated mutants had such defects (*p* < 0.05). This experiment suggests a direct role of increased p53 function in the development of NTDs in *Cited2* mutants which we hypothesize to be mediated through altered acetylation.

## 4 DISCUSSION

In contrast to N‐terminal acetylation, which occurs co‐translationally on most proteins, selected proteins are also subject to post‐translational acetylation of specific lysine residues. The lysine acetylation status of specific proteins depends on a finely tuned equilibrium between histone acetylases (HATs), which catalyze acetylation, and HDACs, which remove acetyl groups (Glozak, Sengupta, Zhang, & Seto, 2005).

Exposure of neurulation‐stage mouse embryos to pharmacological inhibitors of HDACs, such as VPA and trichostatin A (TSA), can cause NTDs as well as skeletal defects (Finnell, Waes, Eudy, & Rosenquist, 2002). Pivotal work in chick embryos in vivo demonstrated that TSA treatment induces NTDs with a complete failure of neural tube closure, accompanied by morphological modifications in neuroepithelial cells and induction of apoptosis (Murko et al., 2013). In previous studies, microarray approaches were used to analyze gene expression alterations in somitic tissues of mouse embryos following VPA exposure (Massa, Cabrera, Menegola, Giavini, & Finnell, 2005). Cluster analysis showed major misregulation of expression in the HDAC ontology group, suggesting a possible role in the subsequent skeletal defects. Modulation of acetylation was hypothesized to mediate the effect of VPA on neurulation, and accordingly increased acetylation of histone H4 in the caudal neural tube was found (Menegola et al., 2005).

Considering the potential clinical relevance of altered acetylation in NTDs and skeletal development, we sought to ascertain the relevance of protein acetylation during spinal cord development in human and mouse embryos. Using different experimental approaches, our study shows a dynamic profile of protein acetylation during the stages of nervous system development in both humans and mice. We also found p53‐acetylation levels to be abnormal in a mouse NTD model, the *Cited2* gene knockout, in which occurrence of NTDs is associated with increased acetylation. p53 was the first nonhistone protein to be shown to be subjected to acetylation modification by HATs and HDACs, with important effects on its stability and consequently on its transcriptional activity (Brooks & Gu, 2011). Importantly, our data are in line with a report of shRNA‐mediated knock‐down of CITED2, resulting in increased acetylated‐p53 in human cancer cells (Wu, Sun, & Chao, 2011). Notably, rescue of the adverse effects of Cited2 loss of function was observed following pifithrin‐α‐mediated inhibition of p53 activity, suggesting a direct role for p53 in causing the exencephaly observed in this model. In conclusion, this study reports a dynamic profile of histone and protein acetylation during CNS development and suggests p53‐acetylation equilibrium as fundamental for primary neurulation in mammals.

## CONFLICT OF INTEREST

The authors declare no conflict of interest.

## Supporting information


**Figure S1** *In vivo* treatments. Schematic representation of pifithrin‐α treatments in the mouse study.

## Data Availability

Data sharing is not applicable to this article as no new data were created or analyzed.
