# Supplementary figures and images for: Dynamic acetylation profile during mammalian neurulation
Source: Birth Defects Res. 2019 Nov 23;112(2):205–11. doi: 10.1002/bdr2.1618 (PMC7004172; doi:10.1002/bdr2.1618)

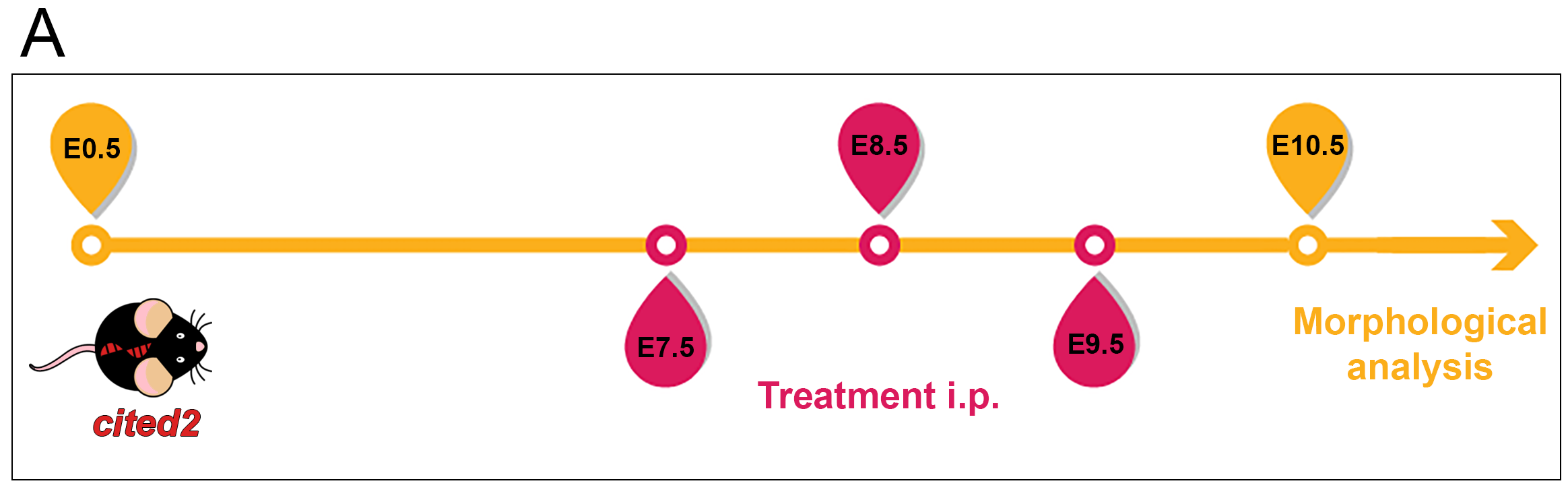

Supplement: Supplementary file 1 — Figure S1 In vivo treatments. Schematic representation of pifithrin‐α treatments in the mouse study. [file BDR2-112-205-s001.tif]
